# Motif-Level Graph Learning Enables Interpretable Prediction of Drug-Induced QT Prolongation via Cooperative Substructural Determinants

**DOI:** 10.3390/ijms27114706

**Published:** 2026-05-23

**Authors:** Wulin Long, Shengqiu Zhai, Yuheng Liu, Menglong Li, Zhining Wen

**Affiliations:** College of Chemistry, Sichuan University, 24 South Section 1, 1st Ring Road, Chengdu 610065, China

**Keywords:** QT prolongation, graph neural networks, interpretable modeling, motif-level representation, pharmacovigilance

## Abstract

Drug-induced QT interval prolongation is a critical safety concern in drug development, yet accurate and mechanistically interpretable prediction from chemical structure remains challenging due to the limited substructural resolution of existing approaches. Here, we present a motif-level graph learning framework for interpretable QT risk prediction. In this framework, molecules are decomposed into chemically meaningful motifs, enabling representation at an intermediate structural scale between atoms and predefined structural alerts. Motif features are encoded using a pre-trained chemical language model, and inter-motif relationships are modeled via attention-based graph learning with cross-scale integration. The model is trained and evaluated on two clinically grounded datasets derived from regulatory drug labeling (DIQTA) and real-world pharmacovigilance data (FAERS), achieving strong and consistent predictive performance with robust generalization across data sources. Importantly, motif-level attention reveals that QT liability is associated with the cooperative organization of compact cationic centers and heteroatom-rich, conformationally adaptable scaffolds, rather than isolated functional groups. These patterns are consistent with known determinants of human ether-à-go-go-related (*hERG*) channel blockade while providing a more structured and chemically specific interpretation beyond conventional structural alerts. Overall, this work establishes a generalizable and interpretable framework for QT risk prediction and highlights motif-level graph learning as an effective strategy for structure-based modeling of adverse drug reactions.

## 1. Introduction

Drug-induced cardiotoxicity remains a major challenge in both clinical pharmacotherapy and drug development. Among its various manifestations, QT interval prolongation is one of the most closely monitored cardiac safety endpoints in regulatory evaluation. The QT interval is an electrocardiographic measure that reflects the duration of ventricular depolarization and re-polarization. Drug-induced QT prolongation generally indicates delayed ventricular re-polarization, which can increase electrical instability of the myocardium and predispose susceptible patients to torsades de pointes and sudden cardiac death [1,2]. Notably, QT prolongation is not limited to drugs targeting the cardiovascular system. Many non-cardiac medications have also been implicated in this liability, often leading to late-stage attrition, regulatory restriction, or post-marketing withdrawal [3,4,5]. Therefore, reliable identification of QT prolongation risk is essential for improving patient safety and informing both drug design and pharmacovigilance.

In recent years, machine learning and deep learning approaches have been increasingly applied to predict QT prolongation and *hERG*-related cardiotoxicity directly from molecular structure [6,7,8,9,10,11]. Current assessment of QT liability, however, still relies predominantly on preclinical assays, including in vitro inhibition of the human ether-à-go-go-related gene (*hERG*) potassium channel and in vivo animal studies [1,12,13]. While these approaches provide valuable mechanistic insights, they are limited by high cost, low throughput, and incomplete translatability to clinical outcomes [14,15,16]. Meanwhile, improved accessibility, curation, and integration of structured regulatory labeling data and large-scale pharmacovigilance databases, such as FAERS, have enabled new opportunities to investigate QT risk from clinically grounded evidence [17,18]. However, these data sources are inherently heterogeneous and often noisy, posing substantial challenges for robust predictive modeling. Moreover, effectively leveraging such heterogeneous clinical data within structure-based predictive frameworks remains a nontrivial task, particularly when balancing predictive performance with interpretability.

Deep learning has enabled significant progress in predicting adverse drug reactions and molecular properties, including toxicity, bioactivity, and pharmacokinetics [19,20,21]. By capturing complex nonlinear relationships between chemical structure and biological outcomes, these models often outperform traditional quantitative structure–activity relationship approaches [22,23]. Benchmark efforts such as MoleculeNet have further standardized evaluation of molecular machine learning models across diverse biochemical tasks [24,25,26]. Nevertheless, limited interpretability remains a critical barrier, particularly in safety-sensitive contexts such as QT prolongation, where mechanistic understanding is essential for regulatory decision-making and medicinal chemistry optimization [27,28,29,30,31]. Despite these advances, existing approaches largely rely on atom-level or sequence-based representations, which are insufficient for capturing higher-order structural organization and cooperative effects among chemically related substructures. Importantly, the challenge of interpretability in QT risk prediction extends beyond model transparency to the level of molecular representation. QT liability typically arises from cooperative effects among multiple substructures that jointly influence ion–channel interactions, lipophilicity, and molecular conformation [1]. For example, protonatable amines embedded within aromatic or heterocyclic scaffolds can contribute synergistically to *hERG* channel blockade [32,33,34]. Atom-level or token-level representations fail to explicitly encode such higher-order structural context and cooperative interactions, often resulting in fragmented or chemically ambiguous explanations. Consequently, existing approaches, including feature attribution on molecular fingerprints, token-level attention on SMILES representations [35], and atom-level graph explanations [31], provide limited actionable insight for chemical design. These limitations highlight the need for molecular representations at an intermediate structural scale that bridge atom-level detail and whole-molecule abstraction. Motif- or fragment-based representations, as explored in junction tree-based generative models and fragment-aware learning frameworks [36,37], provide a natural unit for capturing chemically meaningful substructures. By preserving structural context and functional relevance, such representations offer a promising strategy for improving interpretability while remaining compatible with modern graph-based deep learning architectures [38]. However, their integration into clinically grounded prediction tasks, particularly for complex safety endpoints such as QT prolongation, remains underexplored.

Here, we present a motif-level interpretable graph learning framework for predicting drug-induced QT interval prolongation using clinically grounded data. In this approach, molecules are decomposed into chemically meaningful motifs and represented as graphs to capture interactions among substructures. A graph attention mechanism identifies motifs that contribute most strongly to QT liability, while a cross-attention module integrates complementary global molecular descriptors to enhance predictive performance. The model is trained and evaluated on datasets derived from regulatory drug labeling and pharmacovigilance sources, enabling learning from both curated and real-world evidence [18]. Importantly, although *hERG* channel blockade represents a major mechanistic contributor, QT prolongation is a multifactorial phenotype; the use of clinically grounded datasets therefore facilitates modeling beyond single-channel effects [39]. Specifically, this work makes three main contributions: (i) it introduces a motif-level molecular representation that captures cooperative substructural interactions relevant to QT liability; (ii) it develops an interpretable graph neural network architecture that integrates local motif interactions with global molecular context; and (iii) it establishes a clinically grounded modeling framework by leveraging both regulatory and pharmacovigilance data sources. In addition, recent efforts integrating real-world data and deep learning further support the feasibility of pharmacovigilance-driven modeling strategies. Collectively, this work establishes a clinically relevant and interpretable framework that links chemical structure to QT risk at the sub-structural level, providing both predictive capability and mechanistic insight. More broadly, it offers a generalizable strategy for structure-based and interpretable modeling of adverse drug reactions in complex real-world settings.

In this study, we present a motif-level graph learning framework for interpretable prediction of drug-induced QT prolongation from molecular structure. Unlike atom-level or whole-molecule representations, the proposed framework represents molecules at an intermediate substructural scale, allowing chemically meaningful motifs and their cooperative relationships to be explicitly modeled. By integrating motif-level structural information with global molecular descriptors, the model aims to improve both predictive performance and interpretability.

## 2. Results

In this study, the proposed model predicts QT-positive versus QT-negative status from molecular structure and outputs a QT-positive probability score. Molecules were decomposed into chemically meaningful motifs and represented as motif graphs. Motif embeddings were initialized using MoLFormer and processed by a GATv2-based graph encoder with GRU-based refinement. The learned motif-graph representation was fused with molecular fingerprint features through cross-attention for final prediction. Attention-derived motif scores were used only for post hoc interpretation, and *hERG*-related knowledge was used only as biological context rather than model input or supervision.

### 2.1. Structural Alert Enrichment Associated with QT Prolongation

Structural alert (SA) analysis was conducted to facilitate post hoc chemical interpretation of QT prolongation predictions. Using the ToxAlert module implemented in the OCHEM platform, statistically enriched SAs were identified separately for QT-prolonging and non-QT-prolonging drugs in both the DIQTA and FAERS datasets. Given the higher annotation reliability and regulatory curation of the DIQTA dataset, subsequent mechanistic interpretation primarily focused on DIQTA-derived results.

In the DIQTA dataset, a total of 602 and 669 unique SAs were identified in QT-prolonging and non-QT-prolonging drugs, respectively. Statistical association analysis (χ^2^ test or Fisher’s exact test) identified a subset of alerts significantly enriched in QT- prolonging drugs (*p* < 0.01). A curated selection of representative enriched SAs is summarized in Table 1.

The enriched alerts were predominantly associated with amine-containing substructures, aromatic moieties, and heterocyclic scaffolds. In particular, tertiary amines, general amine functionalities, and nitrogen-containing saturated heterocycles were among the most significantly overrepresented features. These substructures are characteristic of basic and lipophilic drug-like compounds, consistent with known structural determinants of *hERG* channel interaction. In contrast, SAs enriched in non-QT prolonging drugs were more frequently associated with simpler aliphatic frameworks and lacked strongly basic or polyaromatic features.

In the FAERS dataset, a larger number of statistically significant alerts were identified. Among QT signal-positive drugs (*n* = 209), enriched alerts were again dominated by amine-containing substructures, aromatic systems, and nitrogen-rich descriptors (*p* < 0.01). Additional enrichment was observed for halogenated aromatic motifs and six-membered heterocycles. Conversely, QT signal-negative drugs were significantly associated with polar or acidic functionalities, including carboxylic acids, phenols, and aliphatic alcohols. Compared those from DIQTA, alerts derived from FAERS included more chemically non-specific descriptors, reflecting the increased heterogeneity and noise inherent to pharmacovigilance-based labeling. Complete SA analysis results for both datasets are provided in the Supplementary Information.

### 2.2. Model Performance in Predicting Drug-Induced QT Prolongation

#### 2.2.1. Model Performance on DIQTA and FAERS Datasets

Model performance was evaluated on two independently constructed datasets derived from regulatory annotations (DIQTA) and post-marketing pharmacovigilance signals (FAERS). For each dataset, compounds were split into training, validation, and test sets at a 7:2:1 ratio using Bemis–Murcko scaffold-based splitting, and performance metrics were calculated on the held-out test set. Across both datasets, the model demonstrated strong discriminative ability, indicating effective learning of QT-relevant structural and pharmacological features under distinct labeling paradigms.

On the FAERS dataset, the model achieved near-saturated performance, with an AUROC of 0.991 ± 0.004 and an AUPRC of 0.994 ± 0.002. High recall (0.941 ± 0.020), precision (0.941 ± 0.019), and F1 score (0.941 ± 0.016) were consistently observed. On the DIQTA dataset, performance remained robust but was comparatively lower, with an AUROC of 0.976 ± 0.014 and an AUPRC of 0.985 ± 0.010.

All performance metrics were calculated with five independent runs using different random seeds, and results are reported as mean ± standard deviation to ensure statistical robustness. Detailed evaluation metrics are summarized in Table 2.

The high AUROC and AUPRC values indicate that the proposed model achieved strong ranking ability and high precision–recall performance under the present evaluation protocol. This is particularly relevant for QT risk prediction, where early identification of potentially high-risk compounds is important for drug safety assessment. However, these results should be interpreted cautiously. High discriminative performance may partly reflect dataset-specific factors, including chemical class separability, label curation strategy, and residual structural bias despite scaffold-based splitting. This concern is especially relevant for the FAERS-derived dataset, because FAERS labels are based on pharmacovigilance reporting signals rather than confirmed causal QT prolongation. Therefore, the near-saturated FAERS performance may reflect reporting bias and signal amplification in addition to true structure-associated QT liability. The comparatively lower performance on DIQTA may be attributable to its stricter regulatory labeling criteria and more conservative definition of QT risk.

#### 2.2.2. Comparison with Previous QSAR Methods

To further assess the effectiveness of the proposed model, we compared its performance with several previously reported QSAR approaches evaluated on the DIQTA dataset. Since these existing QSAR models were trained using random splitting and five-fold cross-validation, we adopted the same validation strategy in this section to ensure a fair comparison. As shown in Table 3, the proposed model consistently outperformed existing methods across most evaluation metrics. Specifically, our model achieved the highest AUROC (0.946 ± 0.013) and AUPRC (0.967 ± 0.008), indicating superior ranking and early retrieval performance. In addition, the model showed substantial improvement in MCC (0.795 ± 0.030), reflecting better overall balance between sensitivity and specificity.

While some baseline models exhibited competitive recall (e.g., MMGIN), they suffered from reduced specificity and stability, as indicated by large variance values. In contrast, the proposed model maintained a favorable balance between precision (0.917 ± 0.007) and recall (0.928 ± 0.020), resulting in a stable and reliable predictive framework.

### 2.3. Motif Analysis Based on Attention Mechanism

#### 2.3.1. Whole-Dataset Motif Importance Analysis

To interpret the structural basis underlying model predictions, we analyzed motif-level importance derived from the attention mechanism. Attention scores were first averaged across all GATv2 layers and subsequently normalized using intra-molecular Z-score standardization to account for differences in motif counts across molecules.

Motifs with positive Z-scores were defined as high-contribution motifs, and their overall importance was quantified as the average Z-score across all occurrences. This approach avoids arbitrary threshold selection and enables robust comparison across molecular contexts. The top 20 motifs ranked by importance are presented in Table 4.

The distribution of high-contribution motifs revealed a clear enrichment of nitrogen-containing saturated heterocycles and oxygen-containing ring systems. Motifs such as C1CCCNCC1, C1CCCNC1, and C1NCCO1 highlight the importance of protonatable amine centers, which are known to contribute to *hERG* channel binding. A second major group consisted of ether-containing motifs, including cyclic ethers and extended polyether chains (e.g., C1CCOCC1 and C1COCC1), which introduce conformational flexibility and polarity. Aromatic and pseudo-aromatic fragments (e.g., c1cCCCC1, C1 = CCOC1, c) were also prominently represented, suggesting a role for hydrophobic and π-type interactions. Overall, these findings indicate that the model preferentially captures substructures characterized by (i) basic nitrogen atoms, (ii) heteroatom-rich flexible scaffolds, and (iii) aromatic systems, which are consistent with known structural alerts associated with QT prolongation.

#### 2.3.2. Case Study: Motif-Level Interpretation of Representative Drugs

**Figure 1:**
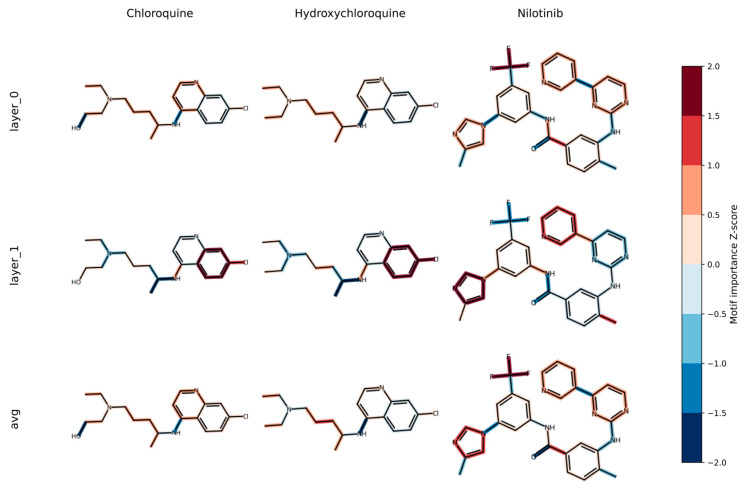
Visualization of motif-level attention for representative QT-prolonging drugs.

To further validate the interpretability of the model, we performed a case study on three representative QT-prolonging drugs: chloroquine, hydroxychloroquine, and nilotinib. As shown in Figure 1, motif-level attention exhibited clear layer-wise differentiation. The first layer preferentially highlighted substructures previously implicated in QT prolongation, while the second layer captured complementary contextual features.

In chloroquine and hydroxychloroquine, high-attention motifs included tertiary amines, heteroaromatic systems (e.g., c1ccncc1), and halogen-substituted aromatic groups (e.g., cCl), all of which are consistent with known *hERG*-binding features. In nilotinib, nitrogen-containing aromatic motifs (e.g., c1cncn1) and electron-withdrawing substituents were prominently identified. Notably, the model also assigned relatively high attention to certain aliphatic chain motifs. This observation suggests the presence of dataset-driven bias, where frequently occurring but non-causal substructures may receive elevated importance.

### 2.4. Result of Ablation Study

To systematically evaluate the contribution of each model component, a series of ablation experiments were conducted. As shown in Table 5, the full model achieved balanced and robust performance, with an accuracy of 0.904 ± 0.022, F1 score of 0.916 ± 0.018, and MCC of 0.813 ± 0.051. All ablation experiments were conducted under the same evaluation protocol as the main model, i.e., 5 independent runs with identical data splits where applicable, ensuring fair comparison.

Removing either modality resulted in noticeable performance degradation. The FP-only model showed reduced predictive power, while the Graph-only model exhibited substantial instability, reflected by large variance in performance metrics. Replacing cross-attention with simple feature concatenation (w/o CA) led to consistent performance decline, demonstrating the importance of explicit cross-modal interaction. The removal of the global node (w/o GN) caused a dramatic drop in performance, indicating that global context aggregation is critical for capturing holistic molecular properties.

Similarly, removing motif-level representation (w/o MG) significantly impaired performance, supporting the importance of substructure abstraction. Excluding MoLFormer embeddings (w/o ME) resulted in marked degradation, highlighting the value of pretrained chemical representations. Finally, removing GRU-based refinement (w/o GRU) led to the most severe performance collapse, suggesting that iterative gated updates are essential for stabilizing representation learning.

## 3. Discussion

Drug-induced QT interval prolongation is a multifactorial clinical phenotype arising from the interplay of molecular structure, physicochemical properties, and biological context. In this study, we demonstrate that motif-level graph representations provide an effective intermediate structural resolution that enables both high predictive performance and mechanistic interpretability for QT liability.

Structural alert analysis was performed as an external validation of model behavior rather than as an input to model training. Across both the regulatory (DIQTA) and pharmacovigilance (FAERS) datasets, QT-prolonging drugs showed consistent enrichment in amine-containing substructures, aromatic systems, and nitrogen-rich heterocycles. These findings are in line with established structural determinants of *hERG* channel blockade. However, the enriched alerts should be interpreted as statistically overrepresented chemical features rather than deterministic indicators of QT liability. For example, although the aromatic alert was observed in 93.5% of QT-prolonging drugs in the DIQTA dataset, it was also present in 76.3% of non-QT-prolonging drugs. This high prevalence in both groups indicates that aromaticity alone has limited discriminative specificity and cannot determine whether a drug causes QT prolongation. Its relevance is more likely context-dependent and becomes chemically informative when aromatic systems co-occur with other QT-associated features, such as protonatable amines, nitrogen-containing heterocycles, ether linkages, halogenated substituents, or conformationally adaptable scaffolds. Notably, the specificity of enriched alerts differed substantially between datasets. Alerts derived from the DIQTA dataset were more compact and chemically well-defined, whereas those from FAERS were broader and included less specific descriptors, reflecting the higher noise level and confounding inherent in spontaneous reporting data. The qualitative agreement between structural alerts and model-derived features supports the chemical plausibility of the learned representations while avoiding circularity, as alerts were not used during model training.

Beyond this coarse-grained analysis, motif-level attention provided a more structured view of QT liability at the substructural level. By averaging attention scores across GATv2 layers and applying intra-molecular Z-score normalization, high-contribution motifs were consistently enriched in nitrogen-containing saturated heterocycles and oxygen-containing ring systems. In particular, motifs corresponding to cyclic amines (e.g., C1CCCNCC1, C1CCCNC1, and C1NCCO1) were among the most highly ranked features. These substructures represent protonatable centers embedded within constrained ring systems, suggesting that the model preferentially captures spatially localized cationic environments rather than merely the presence of basic functional groups. In parallel, a substantial proportion of high-importance motifs consisted of ether-containing and heteroatom-rich cyclic structures (e.g., C1CCOCC1 and C1COCC1). These motifs introduce conformational flexibility and polar linkages within otherwise hydrophobic frameworks, which may facilitate molecular adaptation to the *hERG* channel cavity. Aromatic and heteroaromatic fragments were also identified but were comparatively less dominant in the highest-ranked motifs, indicating that π-mediated interactions may play a secondary role relative to cationic and conformational features in the learned representation. These results suggest that QT liability, as captured by the model, is associated with the co-occurrence of (i) protonatable nitrogen centers, (ii) heteroatom-rich and conformationally adaptable scaffolds, and (iii) auxiliary aromatic features. This pattern is consistent with established structural models of *hERG* binding, in which a cationic center contributes to electrostatic interactions, while surrounding structural elements modulate binding orientation and affinity.

Compared with atom-level attribution or rule-based structural alert systems, motif-level attention provides an interpretable yet expressive representation that captures cooperative substructures and nonlinear interactions. This intermediate resolution avoids excessive fragmentation of chemical context while retaining sufficient granularity to identify chemically meaningful patterns. The observed importance of heteroatom-rich motifs further suggests that QT liability is influenced not only by individual functional groups but also by their structural context and spatial organization.

The strong predictive performance observed on both DIQTA and FAERS datasets further supports the robustness of the proposed framework. The slightly higher performance on the FAERS dataset may reflect the signal amplification and reporting bias inherent in pharmacovigilance data, whereas the more conservative labeling in DIQTA provides a stricter and potentially more challenging benchmark. Importantly, consistent performance across these two datasets indicates that the model captures generalizable features associated with QT risk under different labeling paradigms.

Several limitations should be acknowledged. First, QT prolongation is a complex clinical endpoint influenced by multiple mechanisms beyond *hERG* blockade, including off-target pharmacology and patient-specific factors, which are not explicitly modeled in this study. Second, pharmacovigilance-derived labels are subject to reporting bias and confounding, while the DIQTA and FAERS-derived datasets remain modest in size relative to the complexity of the proposed deep learning architecture. Although scaffold-based splitting, repeated independent runs, and regularization strategies were used to reduce overfitting, the very high AUROC and AUPRC values may still partly reflect dataset-specific chemical separability, label bias, or residual overfitting. Therefore, limited sample size and chemical diversity may affect model generalizability. Third, although attention-based analysis provides useful insights into substructural importance, attention scores reflect model-internal associations and do not establish causal relationships between specific motifs and QT prolongation. These scores may also be influenced by dataset-specific correlations. Fourth, fully independent external validation was not included in the present study. Future work should assess model generalizability using independent hERG datasets, non-overlapping CiPA-like compounds, and temporally separated newly approved drugs with mature QT-related labels.

In summary, this study demonstrates that integrating motif-based graph representations with global molecular descriptors enables accurate and interpretable prediction of drug-induced QT prolongation. By operating at a chemically intuitive yet expressive structural scale, the proposed approach provides a practical framework for early-stage safety assessment and offers mechanistic insights that complement traditional structure-activity analyses.

## 4. Materials and Methods

**Figure 2:**
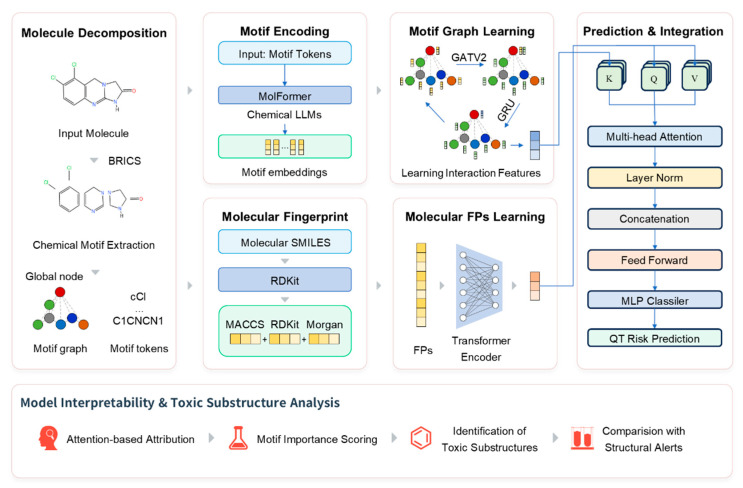
Overview of the motif-level interpretable graph learning framework for predicting drug-induced QT interval prolongation.

The overall workflow of the proposed motif-level graph learning framework is illustrated in Figure 2. The framework was designed to predict drug-induced QT prolongation risk from molecular structure while retaining substructural interpretability. Briefly, each molecule was first decomposed into chemically meaningful motifs using an extended BRICS-based fragmentation strategy. The resulting motifs were encoded by a pretrained chemical language model, MoLFormer [44], to generate motif-level embeddings. These motifs were then organized as a graph, in which nodes represented motifs and edges represented their connectivity within the original molecule. A GATv2-based graph encoder with a global node and GRU-based refinement was used to learn inter-motif interactions [45,46]. In parallel, molecular fingerprints were encoded as global structural descriptors. The motif-graph representation and fingerprint representation were integrated through a cross-attention module, and the fused representation was passed to a prediction head to output the probability of QT-positive classification. Attention weights from the motif graph encoder were further analyzed to identify substructures associated with model predictions.

### 4.1. Data Collection

Two independent datasets were constructed to model drug-induced QT interval prolongation, representing complementary sources of evidence derived from regulatory annotations and post-marketing pharmacovigilance signals. These two datasets capture distinct but complementary aspects of QT liability, namely expert-curated regulatory evidence and real-world reporting signals, respectively, which were developed in parallel and used independently throughout model training and evaluation to assess model robustness across distinct data-generating mechanisms. For each dataset, a scaffold-based splitting strategy was applied to partition the data into training, validation, and test sets at a 7:2:1 ratio. The split was implemented based on Bemis–Murcko molecular scaffolds, with a strict constraint that no overlapping core molecular scaffolds existed among the three subsets, thereby preventing structural information leakage and enabling a more rigorous assessment of model generalization to unseen chemotypes. Separate models were trained and evaluated on the DIQTA and FAERS datasets because the two datasets differ substantially in label definition, data distribution, and underlying data-generating mechanisms. The datasets were not merged into a single training set, as they represent distinct sources of evidence and encode different label semantics. Specifically, DIQTA labels are derived from expert-curated regulatory drug labeling and reflect regulatory assessments of QT-related risk, whereas FAERS labels are based on disproportionality signals from spontaneous adverse event reports and reflect pharmacovigilance reporting associations. Directly combining these datasets would implicitly assume equivalence between regulatory QT-risk categories and reporting-based safety signals, which could introduce label heterogeneity and source-specific bias. Accordingly, the DIQTA and FAERS datasets were modeled, trained, and evaluated independently to assess whether the proposed framework remains robust across distinct evidence sources and data-generating mechanisms.

#### 4.1.1. Regulatory Label–Based Dataset (DIQTA)

The Drug-Induced QT Prolongation Atlas (DIQTA; https://www.adratlas.com/DIQTA/, accessed on 15 December 2025) [47] is a curated resource that classifies marketed drugs according to QT prolongation concern documented in regulatory drug labels. DIQTA provides categorical regulatory annotations rather than continuous QT interval prolongation values. Specifically, drugs are classified into four categories: most concern, moderate concern, ambiguous, and no concern. These annotations are derived from explicit QT-related statements in regulatory labels and reflect expert-curated assessments of drug-induced QT prolongation risk.

In this study, drugs categorized as being of most concern or moderate concern were labeled as QT-prolonging agents, while those annotated as being of no concern were treated as negative controls. Drugs in the ambiguous category were excluded to minimize label uncertainty. Therefore, the model trained on DIQTA predicts categorical QT risk status rather than the actual magnitude of QT interval prolongation. Only small-molecule drugs administered via oral or intravenous routes were retained to ensure pharmacological consistency.

After filtering, the DIQTA dataset comprised 155 QT-positive and 97 QT-negative drugs. This dataset provides high-confidence labels grounded in regulatory assessment and serves as a stringent benchmark for evaluating model performance under well-defined clinical criteria.

#### 4.1.2. Pharmacovigilance Signal-Based Dataset (FAERS)

A second dataset was constructed using the FDA Adverse Event Reporting System (FAERS) [48], a large-scale pharmacovigilance database containing spontaneous reports of adverse drug reactions. FAERS records information such as suspected drugs, reported adverse events, reporting time, and patient-related information when available. However, FAERS does not provide standardized QT interval measurements, confirmed causal QT prolongation outcomes, or continuous QT prolongation time values. Therefore, labels derived from FAERS should be interpreted as pharmacovigilance-signal-based categorical labels rather than as clinically confirmed QT-prolongation endpoints. All reports submitted between 2004 and 2025 were retrieved and standardized. QT prolongation-related adverse events were identified using MedDRA preferred terms corresponding to QT interval prolongation and associated arrhythmogenic conditions. A total of 667 drugs were linked to at least one QT-related report.

To quantify drug-event associations, disproportionality analysis was conducted using the reporting odds ratio (ROR) [49], defined as

(1) $$ROR=\frac{a/c}{b/d}$$

(2) $${SE}_{lnROR}=\sqrt{\frac{1}{a}+\frac{1}{b}+\frac{1}{c}+\frac{1}{d}}$$

(3) $$95\%\;ROR\;Lower\;Bound=\exp\left(lnROR-1.96*{SE}_{lnROR}\right)$$

where *a*, *b*, *c*, and *d* are the numbers of cases defined in Table 6.

A drug was considered QT signal-positive if the lower bound of the 95% confidence interval exceeded 1 [50]. This conservative criterion ensures that only statistically robust disproportionality signals are retained. Based on this criterion, 209 drugs were identified as QT-positive.

Negative controls were defined as drugs without any reported QT-related events across the entire FAERS observation period. To mitigate confounding due to therapeutic class imbalance, negative samples were matched to positive drugs at a 1:1 ratio based on Anatomical Therapeutic Chemical (ATC) classification, yielding 197 matched negatives.

Only orally or intravenously administered small-molecule drugs were retained. QT labels derived from FAERS reflect statistical reporting signals rather than confirmed causality. To improve robustness, conservative signal thresholds and therapeutic class matching were applied to reduce the impact of reporting bias and confounding. This dataset captures real-world safety signals that complement regulatory annotations.

### 4.2. Identification of Structural Alerts

Structural alerts (SAs) were identified to support post hoc chemical interpretation of QT prolongation risk. Structure data files (SDF) were analyzed using the ToxAlert module in the OCHEM platform [51,52].

Alerts enriched in QT-positive and QT-negative classes were extracted separately for the DIQTA and FAERS datasets. Statistical associations were evaluated using the χ^2^ test or Fisher’s exact test, with *p* < 0.01 considered significant. A stringent significance threshold was adopted to enhance the reliability of the findings. Importantly, these analyses were performed solely for interpretability and were not used as model inputs. Representative structural alerts reported in the Results section were further manually curated based on both statistical significance (*p* < 0.01) and chemical interpretability to facilitate mechanistic discussion.

### 4.3. Data Preparation

#### 4.3.1. Molecular Fingerprints

MACCS [53], RDKit, and Morgan [54] fingerprints were generated using RDKit (version 2025.05.6). Morgan fingerprints were computed with radius 4 and length 512 bits. The three fingerprints were concatenated into a 3239-dimensional binary vector, providing complementary structural descriptors at different levels of resolution.

#### 4.3.2. Motif Graph

Molecules were decomposed into chemically meaningful motifs using an extended BRICS-based [36] fragmentation strategy. In addition to standard BRICS rules, two constraints were applied [55]: 1. bonds between ring systems and exocyclic substituents were cleaved; 2. acyclic atoms with ≥3 neighbors were treated as independent motifs. This procedure yielded a motif vocabulary of 12,331 unique substructures (derived from ChEMBL29 [56]), ensuring broad chemical coverage while avoiding excessive fragmentation redundancy. Within the final datasets used in this study, 172 unique motifs were observed in DIQTA and 222 unique motifs were observed in FAERS. On average, each molecule contained 17.31 motifs in DIQTA and 17.59 motifs in FAERS.

While motif abstraction enables modeling of higher-order structural interactions, it removes atom-level detail within motifs. To compensate, each motif was encoded using a pretrained chemical language model (MoLFormer) [44], generating a 768-dimensional embedding that preserves intra-motif chemical semantics. Although some motifs are represented by very short SMILES strings, MoLFormer embeddings can still encode local atom-type, aromaticity, and bonding information learned from large-scale molecular pretraining. For short motifs with limited standalone context, their interpretation relies not only on the MoLFormer embedding itself but also on their connectivity within the motif graph and the global molecular representation.

Each molecule was thus represented as a graph G = (V, E), where nodes correspond to motifs and edges denote motif connectivity. A special [GLOBAL] node was introduced to encode whole-molecule context, connected to all motif nodes, enabling explicit modeling of global structural dependencies.

### 4.4. Model Architecture

#### 4.4.1. Fingerprint Encoding Module

A lightweight Transformer encoder was used to process concatenated fingerprints. The input vector was projected to a 768-dimensional space and processed through 6 Transformer layers (12 heads), producing embedding *e_fp_*, which captures global structural patterns complementary to graph-based representations.

#### 4.4.2. Node Encoding Module

Initial motif features were linearly projected into a shared latent space:

(4) $$h_{v}=LeakyReLU\left(W_{e}x_{v}\right)$$

where $x_{v}$ denotes the original motifs feature vector and $W_{e}$ is a learnable weight matrix. These encoded node embeddings serve as the input to subsequent graph attention layers.

#### 4.4.3. Motif Graph Embedding Module

Graph representations were learned using stacked GATv2 layers with multi-head attention (K = 12). A global node aggregated molecular-level information via a GRU-based update mechanism:

(5) $$h_{v}^{\left(t+1\right)}=\frac{1}{K}\sum_{k=1}^{K}\sum_{u\in N\left(v\right)}\alpha_{vu}^{\left(t,k\right)}W_{k}^{\left(t\right)}h_{u}^{\left(t\right)}$$

(6) $$e_{mol}^{\left(t\right)}=ReLU\left({GRU}_{t}\left(h_{global}^{\left(t\right)},e_{mol}^{\left(t-1\right)}\right)\right)$$

where K (set to 12) is the number of attention heads, N(v) is the neighborhood of node v, $\alpha_{vu}^{\left(t,k\right)}$ is the k-th head attention weight, and $W_{k}^{\left(t\right)}\in R^{D\_hidden\times D\_hidden}$ is the projection matrix. $h_{global}^{\left(t\right)}$ is the global node feature at step t, and $e_{mol}^{\left(t\right)}$ is the updated embedding. The final graph embedding is $e_{graph}=e_{mol}^{\left(T\right)}$, which summarizes hierarchical structural information across the molecule.

#### 4.4.4. Cross-Attention Fusion Module

To integrate graph and fingerprint representations, a cross-attention mechanism was employed:

(7) $$e_{fusion}^{\left(l\right)}=LayerNorm\left(CrossAttn\left(e_{graph},e_{fp},e_{fp}\right)+e_{graph}\right)$$

(8) $$e_{fusion}=LayerNorm\left(FFN\left(e_{fusion}^{\left(l\right)}\right)+e_{fusion}^{\left(l\right)}\right)$$

This mechanism allows dynamic interaction between local motif-level features and global descriptor-based representations, rather than simple feature concatenation.

#### 4.4.5. Prediction Head

The fused representation was passed through a multi-layer perceptron with batch normalization, LeakyReLU activation, and dropout. The final layer outputs a scalar logit, which is transformed by a sigmoid function into a QT-positive probability score.

#### 4.4.6. Attention-Based Motif Importance Analysis

To interpret model predictions at the substructure level, attention scores were extracted from all GATv2 layers and averaged to obtain a unified importance estimate for each motif. To account for varying numbers of motifs across molecules, attention weights were normalized using intra-molecular Z-score standardization. Motifs with Z-scores greater than 0 were defined as high-contribution motifs. The overall importance of each motif was quantified as the average Z-score across all its high-contribution occurrences. Representative drugs used for case studies were selected based on well-documented clinical associations with QT interval prolongation and structural diversity.

### 4.5. Model Evaluation

Model performance was evaluated using multiple metrics, including accuracy, precision, recall rate, F1 score, MCC, AUROC, specificity, and AUPRC.

(9) $$accuracy=\frac{TP+TN}{TP+TN+FP+FN}$$

(10) $$recall\;rate=\frac{TP}{TP+FN}$$

(11) $$precision=\frac{TP}{TP+FP}$$

(12) $$MCC=\frac{TP\times TN-FP\times FN}{\sqrt{\left(TP+FP\right)\left(TP+FN\right)\left(TN+FP\right)\left(TN+FN\right)}}$$

(13) $$F1\;score=\frac{2\times \left(precision\times recall\;rate\right)}{precision+recall\;rate}$$

(14) $$AUROC=\int_{x=0}^{1}TPR\left({FPR}^{-1}\left(x\right)\right)dx$$

(15) $$AUPRC=\int_{-\infty }^{+\infty }precision\left(x\right)dP\left[Y\leq x\right]$$

(16) $$\mathrm{Specificity}=\frac{TN}{TN+FP}$$

TP, TN, FP, and FN stand for true positive, true negative, false positive, and false negative, respectively. To ensure robust evaluation, all metrics were computed on the held-out test set without overlap with training data. Statistical reliability of model performance was assessed using stratified 5 independent runs with different random seeds. All reported performance metrics are presented as mean ± standard deviation across all runs.

### 4.6. Baseline Model

To verify the prediction performance of our proposed model, we selected three representative models in the field of ADR/DIQT prediction as baselines. All baseline models were trained and evaluated under the same data splits, preprocessing pipeline, and evaluation protocol as the proposed model to ensure strict comparability. The baseline models include the following. (i) SVM [42]: A classic structure–activity relationship model using molecular descriptors derived from DIQT-related core structural alerts (amines, ethers, aromatic compounds) as input for DIQT risk prediction. (ii) MoLFormer-XL+CNN Model [40]: A hybrid model leveraging the large-scale pre-trained chemical language model MoLFormer-XL for SMILES feature encoding, combined with a CNN module for downstream ADR classification. (iii) ToxBERT [41]: A task-specific Transformer encoder model based on SMILES sequences, which learns molecular structural features end-to-end via self-attention and masking mechanisms without manual feature engineering, for ADR risk prediction. (iv) MMGIN [43]: A dual-channel multimodal graph isomorphism network for compound toxicity multitask learning, which independently learns molecular representations from compound fingerprints and molecular graphs, and completes downstream prediction via feedforward neural networks with a multitask learning paradigm.

### 4.7. Ablation Study

To rigorously disentangle the contributions of structural representation, initialization strategy, message passing dynamics, and cross-modal interaction, a comprehensive suite of controlled ablation experiments was designed. All ablation variants were evaluated using the same cross-validation strategy and performance metrics as the full model, ensuring consistent and unbiased comparison. Each ablation variant was re-optimized using the same hyperparameter search space as the full model and evaluated under the same data splitting and evaluation protocol.

- Model without [GLOBAL] node (w/o GN). To evaluate the role of explicit global information aggregation, we remove the global node from the motif graph and replace the readout function with a permutation-invariant mean pooling over all motif embeddings.
- Model without motif graph (w/o MG). To evaluate the necessity of motif-level abstraction, the motif graph is replaced by an atom-level graph. Each atom is treated as an individual node and encoded using a 133-dimensional one-hot vector capturing atomic number, degree, formal charge, chiral tag, number of bonded hydrogens, and hybridization state. These features are projected into a 768-dimensional space via a linear layer. The [GLOBAL] node is initialized as the element-wise mean of all atomic node features.
- Model without MoLFormer embeddings (w/o ME). The motif graph structure is retained, while MoLFormer-derived motif node embeddings are replaced by the mean of 133-dimensional projected atomic features within each motif; all other experimental settings are identical to the w/o MG model.
- Model without GRU (w/o GRU). To examine the role of recurrent state refinement, the GRU module is removed. Node representations are updated solely through stacked graph attention (GAT) layers without gated temporal aggregation.
- FPs-only. To quantify the contribution of structural learning from motif graphs, this variant discards the entire motif graph branch and relies exclusively on molecular fingerprint embeddings for QT risk prediction.
- Graph-only. To evaluate the importance of molecular descriptors, the fingerprint branch is removed, and predictions are made solely based on the motif graph representation.
- Model without cross-attention (w/o CA). To assess the effectiveness of cross-modal interaction, the cross-attention module is replaced by a simple concatenation of motif graph and fingerprint embeddings, followed by the same prediction head.

## 5. Conclusions

This study demonstrates that motif-level graph representations provide an effective and chemically interpretable framework for predicting drug-induced QT interval prolongation. By integrating substructural motif features with global molecular descriptors, the proposed model achieves robust predictive performance across both regulatory and pharmacovigilance datasets while maintaining consistent generalization. Importantly, motif-level attention reveals that QT liability is not driven by isolated functional groups, but by the cooperative organization of compact cationic centers and heteroatom-rich, conformationally adaptable scaffolds. These findings refine conventional structural alert paradigms and offer a more mechanistically grounded view of *hERG*-related risk. Beyond QT prolongation, this work establishes motif-level graph learning as a generalizable strategy for interpretable adverse drug reaction prediction, enabling a practical balance between predictive accuracy and mechanistic transparency in early-stage drug safety assessment.

## Figures and Tables

**Table 1:** Structural alerts significantly enriched in QT-prolonging drugs in the DIQTA. dataset.

Class	Name	SA	QT-Prolonging Drugs (*n* = 155)	Non–QT-Prolonging Drugs (*n* = 97)	χ^2^	*p* Value
amine	Tertiary amines	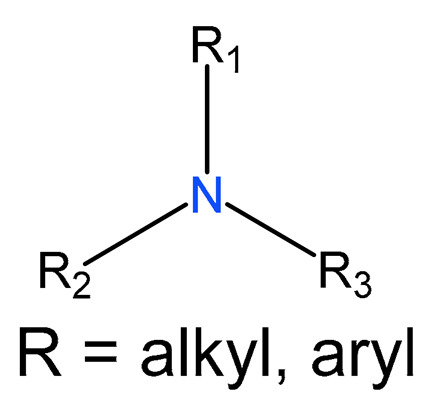	96 (61.9%)	13 (13.4%)	55.30	0.000
	Amines	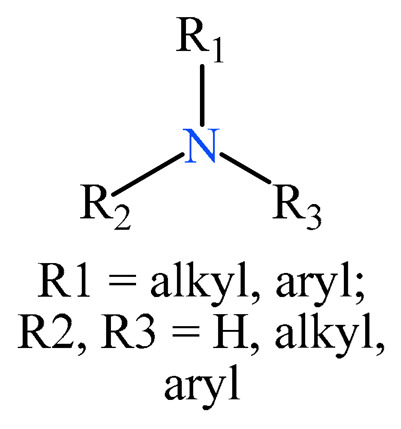	129 (83.2%)	39 (40.2%)	47.77	0.000
	1,2-Diamines	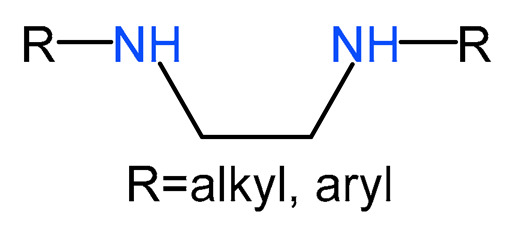	25 (16.1%)	2 (2.1%)	10.91	0.001
heterocycle	Piperidines(HS)	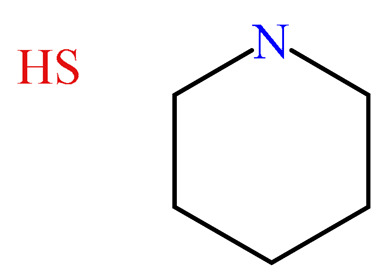	22 (14.2%)	3 (3.1%)	7.03	0.008
	Six-membered heterocycles (HS)	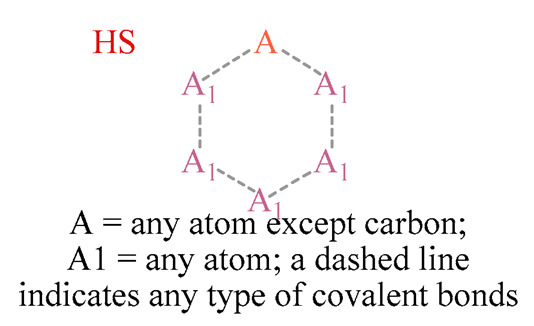	71 (45.8%)	25 (25.8%)	9.32	0.002
	Saturated six-membered heterocycles with one heteroatom (LS)	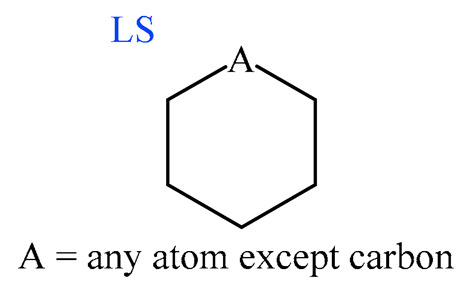	44 (28.4%)	11 (11.3%)	9.19	0.002
Aromatic	Arenes	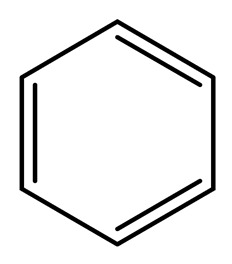	137 (88.4%)	58 (59.8%)	26.26	0.000
	Aromatic	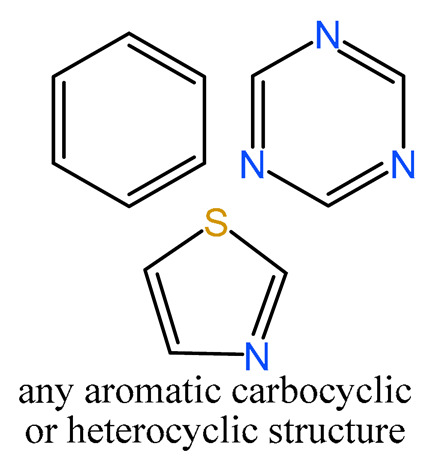	145 (93.5%)	74 (76.3%)	14.14	0.000
Ether	Ethers	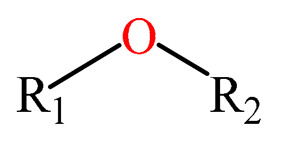	74 (47.7%)	18 (18.6%)	20.68	0.000
	Alkyl aryl ethers	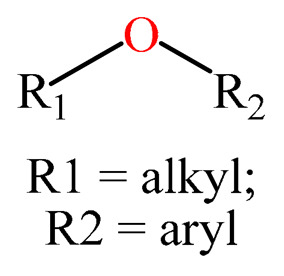	55 (35.5%)	11 (11.3%)	16.76	0.000
Halogen	Halogen derivatives	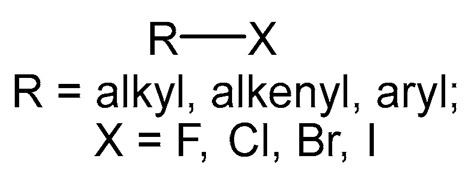	72 (46.5%)	22 (22.7%)	13.42	0.000
	Aryl fluorides	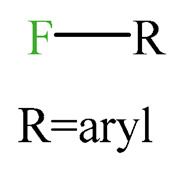	36 (23.2%)	5 (5.2%)	13.01	0.000

**Table 2:** Performance of the proposed model on the DIQTA and FAERS datasets.

Metric	DIQTA	FAERS
Accuracy	0.904 ± 0.022	0.933 ± 0.017
Precision	0.971 ± 0.039	0.941 ± 0.019
Recall	0.867 ± 0.000	0.941 ± 0.020
F1 score	0.916 ± 0.018	0.941 ± 0.016
MCC	0.813 ± 0.051	0.865 ± 0.036
AUROC	0.976 ± 0.014	0.991 ± 0.004
Specificity	0.960 ± 0.055	0.924 ± 0.026
AUPRC	0.985 ± 0.010	0.994 ± 0.002

**Table 3:** Comparison with previously reported QSAR models on the DIQTA dataset.

Metric	MoLFormer-XL-CNN [40]	ToxBERT [41]	SVM [42]	MMGIN [43]	Ours
Accuracy	0.859 ± 0.029	0.783 ± 0.018	0.806 ± 0.014	0.862 ± 0.169	0.903 ± 0.014
AUPRC	0.822 ± 0.079	0.945 ± 0.009	0.791 ± 0.013	0.942 ± 0.071	0.967 ± 0.008
AUROC	0.829 ± 0.060	0.839 ± 0.015	0.790 ± 0.015	0.929 ± 0.087	0.946 ± 0.013
F1 score	0.891 ± 0.022	0.766 ± 0.014	0.844 ± 0.012	0.917 ± 0.102	0.921 ± 0.013
MCC	0.702 ± 0.062	0.604 ± 0.028	0.591 ± 0.031	0.600 ± 0.490	0.795 ± 0.030
Precision	0.845 ± 0.029	0.664 ± 0.034	0.820 ± 0.014	0.862 ± 0.169	0.917 ± 0.007
Recall rate	0.942 ± 0.027	0.913 ± 0.064	0.870 ± 0.017	1.000 ± 0.000	0.928 ± 0.020
Specificity	0.747 ± 0.036	0.700 ± 0.061	0.710 ± 0.026	0.600 ± 0.490	0.859 ± 0.015

**Table 4:** Top 20 motifs ranked by attention-derived importance scores.

Rank	Motifs	z-Score
1	C1CCOCC1	2.2651
2	C1COCC1	2.1018
3	C1C = NCN1	1.8587
4	c1cOCCC1	1.8384
5	c1nCCCC1	1.7674
6	C1NCCO1	1.7569
7	C1SCS1	1.7468
8	C1CCCCCCCCOCCCC1	1.707
9	C1CCCNCC1	1.6629
10	C1CCOC1	1.5951
11	c	1.5547
12	C1 = CCOC1	1.5489
13	cO	1.5232
14	C1CCCOC1	1.4964
15	C1CCCNC1	1.4855
16	C1CCCCCCCCNCCCCO1	1.4722
17	c1cCCCC1	1.4549
18	C1COCS1	1.4537
19	C1COCCC1	1.4532
20	Cc	1.4242

Note. “c” denotes an aromatic carbon atom, and “Cc” denotes an aliphatic carbon connected to an aromatic carbon.

**Table 5:** Results of ablation experiments.

	All	FP-Only	Graph-Only	w/o CA	w/o GN	w/o GRU	w/o ME	w/o MG
Accuracy	0.904 ± 0.022	0.840 ± 0.057	0.856 ± 0.146	0.880 ± 0.028	0.560 ± 0.057	0.44 ± 0.089	0.592 ± 0.128	0.664 ± 0.067
Precision	0.971 ± 0.039	0.901 ± 0.025	0.892 ± 0.167	0.908 ± 0.027	0.811 ± 0.202	0.120 ± 0.268	0.520 ± 0.303	0.688 ± 0.062
Recall	0.867 ± 0.000	0.827 ± 0.121	0.92 ± 0.056	0.893 ± 0.076	0.547 ± 0.425	0.200 ± 0.447	0.760 ± 0.434	0.84 ± 0.192
F1 score	0.916 ± 0.018	0.857 ± 0.062	0.895 ± 0.085	0.898 ± 0.029	0.523 ± 0.236	0.150 ± 0.335	0.610 ± 0.342	0.744 ± 0.071
MCC	0.813 ± 0.051	0.687 ± 0.091	0.689 ± 0.344	0.758 ± 0.055	0.207 ± 0.118	−0.049 ± 0.073	0.133 ± 0.217	0.297 ± 0.171
AUROC	0.976 ± 0.014	0.935 ± 0.017	0.856 ± 0.266	0.956 ± 0.025	0.803 ± 0.16	0.535 ± 0.244	0.701 ± 0.098	0.776 ± 0.03
Specificity	0.960 ± 0.055	0.860 ± 0.055	0.760 ± 0.428	0.860 ± 0.055	0.580 ± 0.531	0.800 ± 0.447	0.340 ± 0.477	0.400 ± 0.274
AUPRC	0.985 ± 0.001	0.948 ± 0.020	0.893 ± 0.208	0.972 ± 0.015	0.858 ± 0.11	0.682 ± 0.146	0.774 ± 0.115	0.820 ± 0.051

**Table 6:** Contingency table for calculating the ROR.

	Cases with Current ADR	Cases Without Current ADR
Cases with current drugs	*a*	*b*
Cases without current ADR	*c*	*d*

## Data Availability

All data and model code are available on GitHub (https://github.com/5HuJiang/motif-based-model, accessed on 13 April 2026).

## Supplementary Materials

The following supporting information can be downloaded at: https://www.mdpi.com/article/10.3390/ijms27114706/s1.

## Abbreviations

The following abbreviations are used in this manuscript:

DIQTA	Drug-Induced QT Prolongation Atlas
FAERS	FDA Adverse Event Reporting System
*hERG*	human ether-à-go-go-related gene
GATv2	Graph Attention Network Version 2
GRU	Gated Recurrent Unit
SA	Structural Alert
OCHEM	Online Chemical Database
MCC	Matthews Correlation Coefficient
AUROC	Area Under the Receiver Operating Characteristic Curve
AUPRC	Area Under the Precision-Recall Curve
