# Supplementary material for: Motif-Level Graph Learning Enables Interpretable Prediction of Drug-Induced QT Prolongation via Cooperative Substructural Determinants
Source: Int J Mol Sci. 2026 May 23;27(11):4706. doi: 10.3390/ijms27114706 (PMC13257067; doi:10.3390/ijms27114706)
Supplement: Supplementary file 1 [file ijms-27-04706-s001.zip › ijms-4286376-supplementary.pdf]

Supplementary Tables

Table S1. FAERS dataset negative drugs.

Index	Drug name	Drugbank_id	ATC code
1	Ethchlorvynol	DB00189	N05CM08
2	Butabarbital	DB00237	N05CB01
3	Methysergide	DB00247	N02CA04
4	Tolcapone	DB00323	N04BX01
5	Trimethadione	DB00347	N03AC02
6	Tacrine	DB00382	N06DA01
7	Profenamine	DB00392	N04AA05
8	Secobarbital	DB00418	N05CB01; N05CA06
9	Methohexital	DB00474	N05CB01; N01AF01; N05CA15
10	Tranylcypromine	DB00752	N06AF04
11	Ethotoin	DB00754	N03AB01
12	Diflunisal	DB00861	N02BA11
13	Anileridine	DB00913	N01AH05
14	Mesoridazine	DB00933	N05AC03
15	Naratriptan	DB00952	N02CC02
16	Ambenonium	DB01122	N07AA30
17	Pemoline	DB01230	N06BA05
18	Isocarboxazid	DB01247	N06AF01
19	Quazepam	DB01589	N05CD10
20	Thioproperazine	DB01622	N05AB08
21	Flunarizine	DB04841	N07CA03
22	Ganaxolone	DB05087	N03AX27
23	Methsuximide	DB05246	N03AD03
24	Brivaracetam	DB05541	N03AX23
25	Salicylamide	DB08797	N02BA75; N02BA55; N02BA05
26	Acetylcarnitine	DB08842	N06BX12
27	Floctafenine	DB08976	N02BG04
28	Tasimelteon	DB09071	N05CH03
29	Idebenone	DB09081	N06BX13
30	Paraldehyde	DB09117	N05CC05
31	Viloxazine	DB09185	N06AX09
32	Lasmiditan	DB11732	N02CC08

Index	Drug name	Drugbank_id	ATC code
33	Brexanolone	DB11859	N06AX29
34	Edaravone	DB12243	N07XX14
35	Samidorphan	DB12543	N05AH53
36	Melitracen	DB13384	N06CA02; N06AA14
37	Medazepam	DB13437	N05BA03
38	Veralipride	DB13523	N05AL06
39	Propyphenazone	DB13524	N02BB74; N02BB54; N02BB04
40	Ethenzamide	DB13544	N02BA07; N02BA77; N02BA57
41	Oxiracetam	DB13601	N06BX07
42	Bornaprine	DB13619	N04AA11
43	Solriamfetol	DB14754	N06BA14
44	Oliceridine	DB14881	N02AX07
45	Ubrogepant	DB15328	N02CD04
46	Atogepant	DB16098	N02CD07
47	Serdexmethylphenidate	DB16629	N06BA15
48	Reserpine	DB00206	C02LA51; C02AA02; C02LA01; C02LA71; C02AA52
49	Methyclothiazide	DB00232	C03AA08; C03AB08
50	Cerivastatin	DB00439	C10AA06
51	Dextrothyroxine	DB00509	C10AX01
52	Clofibrate	DB00636	C10AB01
53	Mecamylamine	DB00657	C02BB01
54	Moricizine	DB00680	C01BG01
55	Moexipril	DB00691	C09AA13; C09BA13
56	Metyrosine	DB00765	C02KB01
57	Hydroflumethiazide	DB00774	C03AA02; G01AE10; C03AH02; C03AB02
58	Fenoldopam	DB00800	C01CA19
59	Conivaptan	DB00872	C03XA02
60	Tocainide	DB01056	C01BB03
61	Deserpidine	DB01089	C02LA03; C02AA05
62	Bretylium	DB01158	C01BD02
63	Polythiazide	DB01324	C03AA05; G01AE10; C03AB05
64	Penbutolol	DB01359	C07CA23; C07AA23
65	Mephentermine	DB01365	C01CA11

Index	Drug name	Drugbank_id	ATC code
66	Oxprenolol	DB01580	C07BA02; C07AA02; C07CA02
67	Rutin	DB01698	C05CA51; C05CA01
68	Prenylamine	DB04825	C01DX52; C01DX02
69	Cyclandelate	DB04838	C04AX01
70	Enoximone	DB04880	C01CE03
71	Nylidrin	DB06152	C04AA02; G02CA02
72	Polidocanol	DB06811	C05BB02
73	Etofibrate	DB08983	C10AB09
74	Acipimox	DB09055	C10AD06
75	Xanthinol	DB09092	C04AD02
76	Levamlodipine	DB09237	C08CA17
77	Bempedoic acid	DB11936	C10AX15; C10BA10
78	Dopexamine	DB12313	C01CA14
79	Zofenopril	DB13166	C09BA15; C09AA15
80	Vincamine	DB13374	C04AX07
81	Buflomedil	DB13510	C04AX20
82	Clopamide	DB13792	C03BB03; C03BA03
83	Fosfructose	DB13863	C01EB07
84	Mavacamten	DB14921	C01EB24
85	Vericiguat	DB15456	C01DX22
86	Finerenone	DB16165	C03DA05
87	Asunaprevir	DB11586	J05AP06; J05AP58
88	Velpatasvir	DB11613	J05AP55; J05AP56
89	Midecamycin	DB13456	J01FA03
90	Grazoprevir	DB11575	J05AP11; J05AP54
91	Sulfametrole	DB15975	J01EE03
92	Cefadroxil	DB01140	J01DB05
93	Ceftizoxime	DB01332	J01DD07
94	Elvitegravir	DB09101	J05AJ02; J05AR09; J05AR18
95	Sparfloxacin	DB01208	J01MA09
96	Cefonicid	DB01328	J01DC06
97	Nafcillin	DB00607	J01CR50; J01CF06
98	Cefiderocol	DB14879	J01DI04
99	Voxilaprevir	DB12026	J05AP56

Index	Drug name	Drugbank_id	ATC code
100	Sulfamethazine	DB01582	J01EB03; G01AE10; J01EE05
101	Relebactam	DB12377	J01DH56
102	Sulfamethizole	DB00576	J01EB02; B05CA04; G01AE10; S01AB01; D06BA04
103	Ticarcillin	DB01607	J01CR50; J01CA13
104	Glecaprevir	DB13879	J05AP57
105	Bictegravir	DB11799	J05AR20
106	Pefloxacin	DB00487	J01MA03
107	Sarecycline	DB12035	J01AA14; J01AA20
108	Oritavancin	DB04911	J01XA05
109	Ceftibuten	DB01415	J01DD14
110	Tecovirimat	DB12020	J05AX24
111	Nirmatrelvir	DB16691	J05AE30
112	Lefamulin	DB12825	J01XX12
113	Ibrexafungerp	DB12471	J02AX07
114	Sulfisoxazole	DB00263	J01EB05; G01AE10; S01AB02
115	Josamycin	DB01321	J01FA07
116	Sulfamerazine	DB01581	J01ED07; G01AE10; D06BA06; J01EE07
117	Cidofovir	DB00369	J05AB12
118	Zanubrutinib	DB15035	L01EL03
119	Teniposide	DB00444	L01CB02
120	Aminoglutethimide	DB00357	L02BG01
121	Alitretinoin	DB00523	L01XF02; D11AH04
122	Upadacitinib	DB15091	L04AA44
123	Mitomycin	DB00305	L01DC03
124	Selumetinib	DB11689	L01EE04
125	Acalabrutinib	DB11703	L01EL02
126	Deucravacitinib	DB16650	L04AA56
127	Copanlisib	DB12483	L01EM02
128	Duvelisib	DB11952	L01EM04
129	Ponesimod	DB12016	L04AA50
130	Treosulfan	DB11678	L01AB02
131	Dactinomycin	DB00970	L01DA01
132	Umbralisib	DB14989	L01EX25

Index	Drug name	Drugbank_id	ATC code
133	Aminolevulinic acid	DB00855	L01XD04
134	Pralsetinib	DB15822	L01EX23
135	Infigratinib	DB11886	L01EN03
136	Pemigatinib	DB15102	L01EN02
137	Pralatrexate	DB06813	L01BA05
138	Fedratinib	DB12500	G01AE10; L01EJ02
139	Diroximel fumarate	DB14783	L04AX09
140	Avacopan	DB15011	L04AA59
141	Diethylstilbestrol	DB00255	L02AA04; L02AA01; G03CC05; G03CB02
142	Pidotimod	DB11364	L03AX05
143	Nelarabine	DB01280	L01BB07
144	Ripretinib	DB14840	L01EX19
145	Pexidartinib	DB12978	L01EX15
146	Idelalisib	DB09054	L01EM01
147	Glycerol phenylbutyrate	DB08909	A16AX09
148	Miglustat	DB00419	A16AX06
149	Odevixibat	DB16261	A05AX05
150	Clebopride	DB13511	A03FA06
151	Cholic Acid	DB02659	A05AA03
152	Obeticholic acid	DB05990	A05AA04
153	Tenapanor	DB11761	A06AX08
154	Lonafarnib	DB06448	A16AX20
155	Isometheptene	DB06706	A03AX10
156	Plecanatide	DB13170	A06AX07
157	Nifuroxazide	DB13855	A07AX03
158	Pipenzolate	DB13844	A03CA09; A03AB14
159	Beta carotene	DB06755	A11CA02; D02BB01
160	Sulbutiamine	DB13416	A11DA02
161	Tiropramide	DB13091	A03AC05
162	Phenolphthalein	DB04824	A06AB04
163	Fenoverine	DB13042	A03AX05
164	Maralixibat	DB16226	A05AX04
165	Ertugliflozin	DB11827	A10BD23; A10BK04; A10BD24
166	Azatadine	DB00719	R06AX09

Index	Drug name	Drugbank_id	ATC code
167	Sobrerol	DB13315	R05CB07
168	Bambuterol	DB01408	R03CC12
169	Bamifylline	DB13203	R03DA08
170	Diphenylpyraline	DB01146	R06AA57; R06AA07
171	Tezacaftor	DB11712	R07AX32; R07AX31
172	Normethadone	DB11609	R05DA06
173	Erdosteine	DB05057	R05CB15
174	Elexacaftor	DB15444	R07AX32
175	Griseofulvin	DB00400	D01BA01; D01AA08
176	Dequalinium	DB04209	D08AH01; R02AA02; G01AC05
177	Thonzylamine	DB11235	D04AA01; R06AC06; R01AC06
178	Abrocitinib	DB14973	D11AH08
179	Verteporfin	DB00460	S01LA01
180	Guanethidine	DB01170	S01EX01; C02CC02; C02LF01
181	Ethylmorphine	DB01466	S01XA06; R05DA01
182	Methscopolamine	DB11315	A03CB01; S01FA03; A03BB03
183	Amodiaquine	DB00613	P01BF03; P01BA06
184	Mebendazole	DB00643	P02CA51; P02CA01
185	Diethylcarbamazine	DB00711	P02CB02
186	Praziquantel	DB01058	P02BA01
187	Trimetrexate	DB01157	P01AX07
188	Tafenoquine	DB06608	P01BA07
189	Artemether	DB06697	P01BF01; P01BE02
190	Phenyl salicylate	DB11071	G04BX12
191	Estetrol	DB12235	G03AA18
192	Lynestrenol	DB12474	G03AB02; G03AA03; G03FA07; G03DC03; G03FB02; G03AC02
193	Oxaprozin	DB00991	M01AE12
194	Tiludronic acid	DB01133	M05BA05
195	Doxacurium	DB01135	M03AC07
196	Deflazacort	DB11921	H02AB13
197	Elagolix	DB11979	H01CC53; H01CC03

Table S2. FAERS dataset positive drugs.

Index	Name	Pubchem_ID	lower 95%CI ROR	ATC code
1	clofazimine	2794	25.95287	J04BA01
2	bepridil	2351	24.58525	C08EA02
3	donepezil	3152	19.24963	N06DA53
4	guanfacine	3519	16.60811	C02AC02
5	tropisetron	656665	14.96337	A04AA03
6	trimebutine	5573	14.96337	A03AA05
7	domperidone	3151	14.14651	A03FA03
8	alfacalcidol	5282181	14.02898	A11CC03
9	bromazepam	2441	12.59861	N05BA08
10	indapamide	3702	10.77371	C03BA11
11	delavirdine	5625	10.43171	J05AG02
12	furosemide	3440	8.786545	C03CA01
13	diphenhydramine	3100	7.882714	D04AA32
14	desloratadine	124087	7.537014	R06AX27
15	imipramine	3696	7.299456	N06AA02
16	gatifloxacin	5379	6.987887	S01AE06，J01MA16
17	pepcid	3325	6.762913	A02BA03
18	(Z)-fuvoxamine	3404	6.684976	N06AB08
19	zuclopenthixol	5311507	6.602649	N05AF05
20	zopiclone	5735	6.229429	N05CF01
21	dexmedetomidine	5311068	6.114631	N05CM18
22	bupropion	444	5.887003	N06AX12
23	bisoprolol	2405	5.677982	C07AB07
24	nortriptyline	4543	5.457997	N06AA10
25	clomipramine	2801	5.391106	N06AA04
26	halofantrine	37393	5.334248	P01BX01
27	nitrendipine	4507	5.220472	C08CA08
28	cimetidine	2756	5.218123	A02BA01
29	bicalutamide	2375	5.097725	L02BB03
30	trimipramine	5584	4.847513	N06AA06
31	aceclofenac	71771	4.676567	M01AB16，M02AA25
32	lopinavir	92727	4.484148	J05AR10

Index	Name	Pubchem_ID	lower 95%CI ROR	ATC code
33	thiabendazole	5430	4.235295	D01AC06
34	chlorpromazine	2726	4.092306	N05AA01
35	clemastine	26987	3.921301	D04AA14
36	linezolid	441401	3.734903	J01XX08
37	sulpiride	5355	3.679741	N05AL01
38	levamisole	26879	3.620379	P02CE01
39	verapamil	2520	3.615134	C08DA01
40	flurazepam	3393	3.568448	N05CD01
41	clevidipine	153994	3.505784	C08CA16
42	propranolol	4946	3.452085	C07AA05
43	lorlatinib	71731823	3.374007	L01ED05
44	cobimetinib	16222096	3.363863	L01EE02
45	triheptanoin	69286	3.300874	A16AX17
46	omeprazole	4594	3.260448	A02BC01
47	acebutolol	1978	3.254151	C07AB04
48	letrozole	3902	3.21379	L02BG04
49	metronidazole	4173	3.205744	D06BX01，J01XD01，A01AB17，G01AF01，P01AB01
50	hydrochlorothiazide	3639	3.147627	C03AA03
51	lincomycin	3000540	3.108071	J01FF02
52	desipramine	2995	3.012487	N06AA01
53	vorinostat	5311	3.001627	L01XH01
54	mazindol	4020	2.992738	A08AA05
55	memantine	4054	2.988737	N06DX01
56	palonosetron	6337614	2.964516	A04AA05
57	oxazepam	4616	2.920457	N05BA04
58	cilostazol	2754	2.900375	B04AC23
59	atenolol	2249	2.653874	C07AB03
60	zonisamide	5734	2.491553	N03AX15
61	sulthiame	5356	2.479466	N03AX03
62	chlorthalidone	2732	2.474076	C03BA04
63	fluphenazine	3372	2.414618	N05AB02
64	tizanidine	5487	2.412549	M03BX02

Index	Name	Pubchem_ID	lower 95%CI ROR	ATC code
65	diltiazem	39186	2.319925	C08DB01，C05AE03
66	amlodipine	2162	2.121808	C08CA01
67	alfuzosin	2092	2.084904	G04CA01
68	sibutramine	5210	2.068058	A08AA10
69	propofol	4943	2.066015	N01AX10
70	lorazepam	3958	2.043157	N05BA06
71	lisdexamfetamine	11597698	2.012306	N06BA12
72	cetirizine	2678	1.976051	S01GX12，R06AE07
73	buspirone	2477	1.92135	N05BE01
74	carfilzomib	11556711	1.899788	L01XG02
75	ceftriaxone	5479530	1.85076	J01DD04
76	zolpidem	5732	1.843151	N05CF02
77	promethazine	4927	1.825044	D04AA10，R06AD02
78	biperiden	2381	1.822719	N04AA02
79	dabrafenib	44462760	1.805322	L01EC02
80	levetiracetam	5284583	1.758184	N03AX14
81	pantoprazole	4679	1.719343	A02BC02
82	carvedilol	2585	1.714109	C07AG02
83	capsofungin	3035406	1.708441	J02AX04
84	metoprolol	4171	1.679116	C07AB02
85	clonidine	2803	1.677836	S01EA04，C02AC01，
86	peramivir	154234	1.621664	J05AH03
87	lansoprazole	3883	1.60344	A02BC03
88	amprenavir	65016	1.591729	J05AE05
89	maprotiline	4011	1.469881	N06AA21
90	triazolam	5556	1.438705	N05CD05
91	nadolol	39147	1.407784	C07AA12
92	dasatinib	3062316	1.359619	L01EA02
93	alprazolam	2118	1.344888	N05BA12
94	tazobactam	123630	1.313256	J01CG02
95	etomidate	667484	1.307646	N01AX07
96	thiethylperazine	5440	1.24682	R06AD03
97	carbamazepine	2554	1.22682	N03AF01
98	methylphenidate	4158	1.214226	N06BA04

Index	Name	Pubchem_ID	lower 95%CI ROR	ATC code
99	decitabine	451668	1.196946	L01BC08
100	epirubicin	41867	1.178348	L01DB03
101	alectinib	49806720	1.165592	L01ED03
102	levocetirizine	1549000	1.133925	R06AE09
103	doxazosin	3157	1.122705	C02CA04
104	mirabegron	9865528	1.11362	G04BD12
105	prazosin	4893	1.097351	C02CA01
106	clonazepam	2802	1.08275	N03AE01
107	milnacipran	65833	1.0745	N06AX17
108	lamotrigine	3878	1.069975	N03AX09
109	doxorubicin	31703	1.066471	L01DB01
110	lacosamide	219078	1.046261	N03AX18
111	loratadine	3957	1.043585	R06AX13
112	chlordiazepoxide	2712	1.021107	N05BA02
113	theophylline	2153	1.019964	R03DA04
114	cisapride	6917698	245.723	A03FA02
115	ibutilide	60753	101.1455	C01BD05
116	bedaquiline	5388906	86.04323	J04AK05
117	doxapram	3156	44.5146	R07AB01
118	sotalol	5253	36.51784	C07AA07
119	vandetanib	3081361	32.04245	L01EX04
120	dofetilide	71329	31.49374	C01BD04
121	procainamide	4913	24.34737	C01BA02
122	methadone	4095	21.33502	N07BC02
123	loperamide	3955	20.08303	A07DA03
124	flecainide	3356	19.53039	C01BC04
125	disopyramide	3114	15.3827	C01BA03
126	amisulpride	2159	14.58444	N05AL05
127	hydroxyzine	3658	13.89899	N05BB01
128	nilotinib	644241	13.64782	L01EA03
129	citalopram	2771	13.5303	N06AB04
130	ziprasidone	60854	13.3862	N05AE04
131	ribociclib	44631912	13.10178	L01EF02
132	azithromycin	447043	13.04603	S01AA26，J01FA10

Index	Name	Pubchem_ID	lower 95%CI ROR	ATC code
133	thioridazine	5452	12.86202	N05AC02
134	amiodarone	2157	12.80166	C01BD01
135	chloroquine	2719	12.37743	P01BA01
136	amitriptyline	2160	12.23627	N06AA09
137	primaquine	4908	12.09317	P01BA03
138	quinidine	441074	11.61254	C01BA01
139	hydroxychloroquine	3652	11.40983	P01BA02
140	ondansetron	4595	11.27569	A04AA01
141	ethionamide	2761171	10.69012	J04AD03
142	haloperidol	3559	10.51904	N05AD01
143	escitalopram	146570	10.38167	N06AB10
144	fluconazole	3365	10.2122	J02AC01，D01AC15
145	gilteritinib	49803313	9.606897	L01EX13
146	glasdegib	25166913	9.441663	L01XJ03
147	midostaurin	9829523	9.16458	L01EX10
148	trazodone	5533	8.638522	N06AX05
149	iloperidone	71360	8.105986	N05AX14
150	propafenone	4932	7.659236	C01BC03
151	posaconazole	468595	7.019678	J02AC04
152	mirtazapine	4205	6.755402	N06AX11
153	clarithromycin	84029	6.676891	J01FA09
154	fluoxetine	3386	6.633533	N06AB03
155	moxifloxacin	152946	6.631972	S01AE07，J01MA14
156	cyproheptadine	2913	6.563477	R06AX02
157	itraconazole	55283	6.36148	J02AC02
158	quinine	3034034	6.304134	P01BC01
159	quetiapine	5002	6.128359	N05AH04
160	osimertinib	71496458	5.999561	L01EB04
161	dronedarone	208898	5.884041	C01BD07
162	droperidol	3168	5.433567	N05AD08
163	ivabradine	132999	5.221049	C01EB17
164	nelfinavir	64143	4.913228	J05AE04
165	venlafaxine	5656	4.619085	N06AX16
166	encainide	48041	4.581108	C01BC08

Index	Name	Pubchem_ID	lower 95%CI ROR	ATC code
167	erythromycin	12560	4.422943	J01FA01, D10AF02, S01AA17
168	ranolazine	56959	4.3286	C01EB18
169	pimozide	16362	4.309282	N05AG02
170	rilpivirine	6451164	4.250562	J05AG05
171	vemurafenib	42611257	4.124923	L01EC01
172	galantamine	9651	4.022897	N06DA04
173	atomoxetine	54841	3.54118	N06BA09
174	sertraline	68617	3.21672	N06AB06
175	crizotinib	11626560	3.195317	L01ED01
176	voriconazole	71616	3.173672	J02AC03
177	risperidone	5073	3.130919	N05AX08
178	levofloxacin	149096	3.081531	J01MA12, S01AE05
179	atazanavir	148192	2.996073	J05AE08
180	ceritinib	57379345	2.948186	L01ED02
181	aripiprazole	60795	2.855933	N05AX12
182	mitotane	4211	2.727881	L01XX23
183	tolterodine	443879	2.620379	G04BD07
184	tamoxifen	2733526	2.51841	L02BA01
185	ciprofloxacin	2764	2.444025	S03AA07, J01MA02, S02AA15
186	toremifene	3005573	2.322558	L02BA02
187	etelcalcetide	71511839	2.246639	H05BX04
188	paroxetine	43815	2.002613	N06AB05
189	ktoconazole hra	3823	1.989797	G01AF11
190	fingolimod	107970	1.980821	L04AA27
191	solifenacin	154059	1.75682	G04BD08
192	saquinavir	441243	1.737006	J05AE01
193	tramadol	33741	1.709607	N02AX02
194	darunavir	213039	1.694936	C07AB12
195	paliperidone	115237	1.65255	N05AX13
196	nebivolol	71301	1.534772	C07AB12
197	spironolactone	5833	1.533396	C03DA01

Table S3. SA analysis results of DIQTA dataset.

SA	count_positive (n=155)	count_negative (n=97)	statistic	p_value
Tertiary amines	96	13	55.29	<0.001
sp3 hybridized carbon atoms (2)	126	36	48.80	<0.001
Amines	129	39	47.77	<0.001
16-Tertiary amine	100	19	46.53	<0.001
Tertiary aliphatic amines	83	10	46.06	<0.001
B3-tertiary amine	83	10	46.06	<0.001
Nitrogen atoms (1)	85	12	43.67	<0.001
BASE	137	51	38.51	<0.001
36-CH2N (16-Tertiary amine)	73	14	26.73	<0.001
Arenes	137	58	26.25	<0.001
Ethers	74	18	20.68	<0.001
2-CH2 (1-Alkane group)	140	65	19.86	<0.001
48-CH2S	3	17	17.77	<0.001
sp3 hybridized carbon atoms (4)	3	17	17.77	<0.001
Alkyl aryl ethers	55	11	16.76	<0.001
Aryl halide	59	13	16.59	<0.001
Aryl halides	59	13	16.59	<0.001
Aromatic Halogen	58	13	15.84	<0.001
Six-membered heterocycles with one heteroatom (LS)	74	22	14.84	<0.001
10-ACH (3-Aromatic carbon)	140	69	14.19	<0.001
C, N, O, P and S atoms in unusual valence states	145	74	14.13	<0.001
Aromatic	145	74	14.13	<0.001
35-CH3N(16-Tertiaryamine)	37	5	13.73	<0.001
Four-membered heterocycles with one heteroatom (LS)	2	13	13.54	<0.001
Saturated four-membered heterocycles with one heteroatom (LS)	2	13	13.54	<0.001
Four-membered heterocycles (LS)	2	13	13.54	<0.001
13-Ether	72	22	13.41	<0.001
Halogen derivatives (alkyl, alkenyl, aryl)	72	22	13.41	<0.001

SA	count_positive (n=155)	count_negative (n=97)	statistic	p_value
Halogens	72	22	13.41	<0.001
Aryl fluorides	36	5	13.00	<0.001
4-Aromatic carbon-alkane	90	33	12.85	<0.001
3-Aromatic carbon	141	71	12.81	<0.001
38-Aromatic fluoro	35	5	12.29	<0.001
71-ACF (38-Aromaticfluoro)	35	5	12.29	<0.001
Tertiary Amine	42	8	12.16	<0.001
sp3 hybridized carbon atoms (6)	93	36	11.60	<0.001
11-AC (3-Aromaticcarbon)	127	60	11.54	<0.001
Tertiary mixed amines (arylalkyl)	28	3	11.04	<0.001
1,2-Diamines	25	2	10.91	<0.001
Halogenated benzene	42	9	10.65	0.001
Sulphur atom (2)	1	9	9.51	0.002
Six-membered heterocycles (HS)	71	25	9.32	0.002
Saturated six-membered heterocycles with one heteroatom (LS)	44	11	9.18	0.002
1-Alkanegroup	148	81	8.92	0.002
103-CH2S(48-CH2S)	2	10	8.80	0.003
14-ACCH(4-Aromaticcarbon-alkane)	51	15	8.50	0.003
α,β-Unsaturated carbonyl and thiocarbonyl	1	8	7.92	0.004
Unsaturated six-membered heterocycles with two heteroatoms (LS)	6	14	7.72	0.005
sp2 hybridized carbon atoms (11)	147	81	7.62	0.005
Nitrogen atoms (4)	43	12	7.38	0.006
Pnictogens(nitrogen group)	153	88	7.30	0.006
Saturated six-membered heterocycles with two heteroatoms (LS)	32	7	7.23	0.007
Piperidines (HS)	22	3	7.03	0.008
Hexahydro diazines	27	5	7.02	0.008
Aromatic five-membered heterocycles with one hetero atom (LS)	16	1	6.77	0.009

Table S4. SA analysis results of FAERS dataset.

SA	count_positive (n=209)	count_negative (n=197)	statistic	p_value
sp3 hybridized carbon atoms (2)	154	91	30.89	<0.001
BASE	169	108	30.53	<0.001
Tertiary amines	105	46	30.25	<0.001
10-ACH (3-Aromaticcarbon)	196	147	26.96	<0.001
Pnictogens (nitrogen group)	204	163	24.13	<0.001
Amines	154	98	23.68	<0.001
3-Aromaticcarbon	196	150	23.67	<0.001
C, N, O, P and S atoms in unusual valence states	197	152	23.18	<0.001
Aromatic	197	152	23.18	<0.001
Tertiary aliphatic amines	91	41	22.85	<0.001
B3-tertiaryamine	91	41	22.85	<0.001
Nitrogen atoms (1)	94	44	22.17	<0.001
Arenes	186	139	20.45	<0.001
sp2 hybridized carbon atoms (11)	200	164	15.62	<0.001
11-AC(3-Aromaticcarbon)	171	129	13.19	<0.001
Tertiary Amine	50	21	11.46	0.001
A1-e.g., carboxylic acid	12	32	10.51	0.001
Aryl halide	78	44	10.13	0.001
Aryl halides	78	44	10.13	0.001
Aromatic Halogen	76	43	9.65	0.002
Tertiary mixed amines (arylalkyl)	34	12	9.47	0.002
Halogenated benzene	59	30	9.27	0.002
ACID	29	52	9.19	0.002
Aryl chlorides	52	25	9.03	0.003
16-Tertiaryamine	111	75	8.64	0.003
Aliphatic alcohols	1	12	8.58	0.003
25-Aromaticchloro	51	25	8.39	0.004
54-ACCl(25-Aromaticchloro)	51	25	8.39	0.004
2-Olefingroup	31	53	8.28	0.004
ELEC	40	63	8.17	0.004
4-chlorobenzene	49	24	7.98	0.005
E3-e.g., carbonates	44	67	7.93	0.005

SA	count_positive (n=209)	count_negative (n=197)	statistic	p_value
1-CH3(1-Alkanegroup)	104	126	7.76	0.005
1,2-Diamines	27	9	7.75	0.005
Anilines, anilides	47	23	7.57	0.006
Six-membered heterocycles (HS)	84	53	7.43	0.006
Phenols	7	21	7.34	0.007
8-Aromaticcarbon-alcohol	7	21	7.34	0.007
18-ACOH(8-Aromaticcarbon-alcohol)	7	21	7.34	0.007
A33-phenol	7	21	7.34	0.007
Hexahydro diazines	28	10	7.32	0.007
Carboxy licacids	12	28	7.27	0.007
20-COOH	12	28	7.27	0.007
E4EXC	12	28	7.27	0.007
Halogen derivatives (alkyl, alkenyl, aryl)	86	55	7.26	0.007
Halogens	86	55	7.26	0.007

Table S5. Input features, model size, and training settings of models compared on the DIQTA dataset.

Model	Trainable parameters	Input features	Data split	Optimizer	Learning rate setting	Max epoch	Early stopping
Ours	37,406,882	Motif graph with MolFormer motif embeddings and molecular fingerprints	Five-fold cross-validation	Adam	Initial LR = 1e-3; min LR = 1e-8	200	Patience = 10
MolFormer-XL-CNN	47,963,137	SMILES sequence	Five-fold cross-validation	Adam	Initial LR = 1e-3; min LR = 1e-8	200	Patience = 10
ToxBERT	3,581,246	SMILES sequence	Five-fold cross-validation	Adam	Initial LR = 1e-3; min LR = 1e-8	200	Patience = 10
MMGIN	1,733,121	Molecular fingerprints and atom-level molecular graph	Five-fold cross-validation	Adam	Initial LR = 1e-3; min LR = 1e-8	200	Patience = 10
SVM	Not applicable	Molecular fingerprints	Five-fold cross-validation	Not applicable	Not applicable	Not applicable	Not applicable

Table S6. Attention based Z-score for motifs in the FAERS dataset.

Rank	Motifs	Z-score
1	C1CNC[C@@H]2CSSCCCNCCNCCN[C@H](CNCCNCCNCCNCCN2)CSSCC NCCN1	3.30
2	C1COCC1	2.66
3	C1CCCS1	2.62
4	C1CCNCN1	2.60
5	c1nccoc-1	2.49
6	C1COCCC1	2.40
7	C1CCCNCCC1	2.39
8	C1CC2CCC1C2	2.29
9	c1cnnn1	2.25
10	c1c[nH]cn1	2.23
11	c1cocn1	2.23
12	c1cnc[nH]c1	2.19
13	cCl	2.16
14	c1cCOC1	2.12
15	C1CCCCCCCC/C=C/C=C/CCO1	2.10
16	C1NCCN2C[C@@H](CC2)OccC/C=C/COCCO1	2.10
17	cBr	2.09
18	c1cncn1	2.07
19	C1CCCOC1	2.06
20	C1Cc2cc3ccc(cc4nc(cc5ccc(cc1n2)[nH]5)C=C4)[nH]3	2.03

Table S7. Features of the atom graph.

**Feature**	**Description**	**Feature dimension**
Atomic number	0–99, corresponding to atomic numbers 1–100	101
Atom degree	0, 1, 2, 3, 4, 5	7
Formal charge	−2, −1, 0, 1, 2	6
Chirality tag	0, 1, 2, 3	5
Number of bonded hydrogen atoms	0, 1, 2, 3, 4	6
Hybridization type	SP, SP2, SP3, SP3D, SP3D2	6
Aromaticity	Yes = 1, No = 0	1
Atomic mass	Relative atomic mass × 0.01	1

Note: Except for aromaticity and atomic mass, one additional dimension was added to each featu re to represent unknown values.
